# A Sequential Study on the Pathology of Peste Des Petits Ruminants and Tissue Distribution of the Virus Following Experimental Infection of Black Bengal Goats

**DOI:** 10.3389/fvets.2021.635671

**Published:** 2021-02-19

**Authors:** Shahana Begum, Mohammed Nooruzzaman, Mohammad Rafiqul Islam, Emdadul Haque Chowdhury

**Affiliations:** Department of Pathology, Faculty of Veterinary Science, Bangladesh Agricultural University, Mymensingh, Bangladesh

**Keywords:** PPRV, lineage IV, Bangladesh, pathology, Black Bengal goats

## Abstract

We studied the sequential pathology of peste des petits ruminants (PPR) in Black Bengal goats and analyzed virus distribution in tissues and virus shedding following experimental infection with a Bangladeshi isolate of lineage IV PPR virus (PPRV). The early clinical signs like fever, depression, and ocular and nasal discharges first appeared at 4–7 days post-infection (dpi). Three out of eight inoculated goats died at 13, 15, and 18 dpi, and the rest were killed at different time points from 5 to 18 dpi. Initially, the virus multiplied mostly in the lymphoid organs of the pharyngeal region and caused extensive lymphoid destruction and hemorrhages. This was followed by viremia, massive virus replication in the lungs, and pneumonia along with the appearance of the clinical signs. Subsequently, the virus spread to other organs causing necrotic and hemorrhagic lesions, as well as the virus localized in the upper respiratory, oral and intestinal mucosa resulting in catarrhal, erosive, and ulcerative lesions. On hematological and biochemical investigation progressive leukopenia and hypoproteinemia, a gradual increase of serum metabolites and enzymes associated with liver and kidney damage, and electrolyte imbalance were observed. Seroconversion started at 7 dpi and all the surviving animals had serum antibodies at 14 dpi. Virus shedding was observed in nasal and ocular secretions at 4 dpi and in feces and urine at 14 dpi, which gradually increased and continued till the end of the experiment (18 dpi) despite seroconversion. Therefore, the virus shedding of naturally infected seroconverted goats should be monitored for effective control strategies.

## Introduction

Peste des petits ruminants (PPR), also known as goat plague, is a disease of goats, sheep, and other taxonomically related species. It is caused by PPR virus (PPRV), a morbillivirus under the family *Paramyxoviridae*, and genetically related to rinderpest virus (RPV), measles virus (MV), and canine distemper virus (CDV) (1). PPRV carries a single-stranded RNA genome of 15,948 bp size; that encodes for 6 structural proteins, including a nucleoprotein (N), a viral RNA-dependent polymerase (L), an RNA-polymerase phosphoprotein co-factor (P), a matrix protein (M), a fusion protein (F) and a hemagglutinin protein (H) and two non-structural proteins (C and V proteins) due to RNA editing of the phosphoprotein gene (2). Although only one serotype of PPRV is known to exist, phylogenetic studies indicate that PPRV strains can be divided into four distinct lineages. The lineages correlate well with geographic distribution of the virus, with lineages I and II mainly restricted to western and central Africa, lineage III to eastern Africa and the Arabian Peninsula, and lineage IV to Southeast Asia, the Middle East, and more recently northern, western, central and eastern regions of Africa and is gradually moving southwards (3–6).

The PPRV transmits to the susceptible animals through direct contact with infected animals via secretions or feces (7). The virus has special affinity to lymphoid tissues and replication of the virus in lymphoid tissues causes extensive necrosis of lymphocytes leading to leukopenia (8–10). Besides, PPRV also replicates extensively in epithelial cells leading to necrohemorrhagic changes in different visceral organs. Virus replication and dissemination in the epithelial cells causes necrosis leading to conjunctivitis, necrotizing and erosive stomatitis and enteritis and death in fatal courses due to severe bronchopneumonia and/or severe diarrhea and dehydration (10–14).

The pathology of PPR has been shown to vary across species, breeds and age of the susceptible animals as well as the genetic composition of the virus. For instance, goats are considered more susceptible than sheep and outbreaks in goats tended to be more severe (13, 15–18). Similarly, a different disease severity has been observed in different breeds of goats (8, 10–14, 19). Moreover, a variation in the mortality and clinical disease has been observed in goats upon infection with PPRV of different lineages (11, 20).

Black Bengal goat is a popular breed in Bangladesh and reared in both rural households and urban areas of Bangladesh. There were about 26.1 million goats in Bangladesh in the year 2017–2018 and 41% farm incomes came from goats in some parts of Bangladesh (21). The outbreak of PPR in Bangladesh was first recognized in 1993 (22). PPR is now endemic in Bangladesh causing huge economic losses every year. Studies of PPR in Bangladesh have mostly been limited to seromonitoring and risk factor analysis of the disease, molecular epidemiological investigation of the virus, and gross and histopathological investigation of PPR affected dead goats (4, 5, 12, 23, 24).

After successful eradication of the rinderpest of cattle, the Food and Agricultural Organization (FAO) and the World Animal Health Organization (OIE) have targeted the global eradication of PPR. Bangladesh has joined the “PPR Global Eradication Program 2015–2030”. Successful eradication of any disease requires an in-depth understanding of the pathobiology of the disease. Few studies have addressed the sequential pathology of PPR in goats upon experimental infection (13, 14, 18, 19). However, the sequential pathology of PPR and virus distribution in tissues and virus shedding in Black Bengal goats have not been studied yet. Hence we performed a sequential study on the pathology of PPR in Black Bengal goats and analyzed the distribution of the virus in tissues and shedding of the virus in nasal and ocular secretions, feces and urine following experimental infection with a lineage IV PPRV isolate from Bangladesh. The findings of this study would help in a better understanding of the pathobiology of PPR for the formulation of effective national PPR control and eradication plans in Bangladesh and elsewhere.

## Materials and Methods

### Ethics Statement

All applicable national and institutional guidelines for the care and use of animals were followed. The study was carried out in accordance with the recommendation of the “Ethical Standard of Research Committee” of Bangladesh Agricultural University, Mymensingh. The protocol and procedures employed were reviewed and approved by the “Ethical Standard of Research Committee” (Ref. No. BAURES/ESRC/699/2020; Dated: 28.06.2020). According to the 3R principle (Replace, Reduce and Refine) of animal experimentation, the number of experimental animals was kept to minimal. Handling of live animals was performed by expert veterinarian. Infected goats that were unable to stand, eat or drink were sacrificed to reduce the suffering.

### Animals

A total of 12 healthy Black Bengal goats aged between 4 and 8 months were purchased from local markets. The animals had no history of previous PPR vaccination or exposure to clinical PPR infection. Animals were kept in relative isolation for about 2 weeks with adequate feed and water. Routine deworming was performed using subcutaneous Ivermectin injection as per instruction. Before animal experimentation, all goats were tested for anti-PPRV antibodies with a commercial competitive ELISA kit (ID Screen PPR Competition, ID-Vet, Montpellier, France) and found seronegative.

### Virus and Cell Culture

A local strain of PPRV (BD/PPRV/2015/1) classified under lineage IV was used for the experimental infection. The PPRV strain (BD/PPRV/2015/1) was originally collected from a field outbreak in Black Bengal goats in 2015 (10) and subsequently isolated in primary goat kidney cells. The PPRV inoculum was prepared using primary goat kidney cell culture (25) and the presence of virus in the culture supernatant was tested by real-time quantitative RT-PCR (qPCR) as described previously (26). Finally, the amount of virus in the culture supernatant was titrated using 96 well flat bottom tissue culture plates (27) and the end-point titer was calculated by Reed and Muench method (28).

### Experimental Infection

On the day of infection, animals were divided into two groups: infected (*n* = 8) and control (*n* = 4) groups and housed separately. Each goat of the infected group was inoculated with 2 mL of the PPRV inoculum having a titer of 5.6 log_10_ TCID_50_/mL through the intranasal route. Control goats received 2 mL sterile PBS per animal via the same route. Both the infected and control goats were observed daily for feed intake, rise in body temperature and development of clinical signs and oral lesions. A predefined endpoint of the experimental study at 18 days post infection (dpi) was considered assuming the severity of the clinical disease in the infected goats.

### Sample Collection

Blood samples were collected at 0, 5, 7, 10, 14, and 18 days post-infection (dpi) from both infected and control goats. Immediately after collection, 2 mL of the blood sample was transferred to a sterile tube containing ethylenediaminetetraacetic acid (EDTA) at 1 mg/mL blood for routine hematological examination. Besides, 5 mL of the blood sample was transferred to a tube without EDTA for serum separation. Swab samples from different natural opening (nasal, ocular, urine and feces) of both infected and control goats were collected in a virological transport medium at 0, 3, 4, 5, 7, 14, 18 dpi and stored at −80°C. One PPRV-infected goat was sacrificed at each time point of 5, 7, 10, 14, and 18 dpi. In addition, one control goat was also sacrificed at each time point of 5, 7, 14, and 18 dpi. Routine necropsy was performed following strict biosafety measures and gross lesions were recorded. Necropsy was also performed on animals that died of PPR infection. Tissue samples from the lips, trachea, lungs, spleen, lymph node, intestine, kidney, heart, spleen and liver were collected in sterile tubes for virus quantification and stored at −80°C. Tissue samples from different organs were also collected in 10% neutral buffered formalin for histopathological and immunohistochemical studies.

### Competitive Enzyme-Linked Immunosorbent Assay (cELISA)

A competitive ELISA kit (ID Screen PPR Competition, ID-Vet, Montpellier, France) was used to measure the amount of PPRV specific antibodies in sera of PPRV-infected and healthy goats following the protocol recommended by the manufacturer. As mentioned in the kit, a sample having a competition percentage (CP) value of ≤ 35 was considered as seropositive.

### Hematobiochemical Analysis

Routine hematological examination of whole blood samples collected at different time points post-infection was performed by the standard method (29). Routine hematological parameters such as hemoglobin, erythrocyte sedimentation rate, packed cell volume, total erythrocyte count, total leucocyte count and differential leukocyte count were measured. In addition, different serum biochemical constituents such as total protein, albumin, glucose, bilirubin, blood urea nitrogen, creatine kinase, alkaline phosphatase, alanine transaminase and aspartate transaminase, inorganic phosphorus and calcium were analyzed using an automated T80 Ultraviolet-visible spectroscopy (UV/VIS) spectrophotometer (PG Instruments, UK). Furthermore, an automated electrolyte analyzer GENLYTE 3000A (IVD) was used to analyze serum electrolytes (sodium, potassium, and chloride ions) using a commercial kit (Electrolyte solution, Biogen, GmbH, Germany).

### Histopathology

Formalin-fixed tissues were processed and embedded with paraffin. A 5 μm section was prepared from paraffin block and then stained with routine hematoxylin and eosin (H&E) following the standard procedure (30).

### Quantification of PPRV RNA in Different Tissues and Secretions

The amount of PPRV RNA in different tissues of the infected goats collected at 5, 7, 14, and 18 dpi were quantified using real-time quantitative RT-PCR (qPCR). In addition, virus shedding in nasal and ocular secretions, urine and feces was quantified using qPCR at 0, 3, 4, 5, 7, 14, and 18 dpi. Briefly, a 20% tissue homogenate was prepared in sterile PBS. Total RNA was extracted with PureLink RNA Mini Kit (ThermoFisher Scientific, Waltham, MA, USA) and quantified by NanoDrop 2000 spectrophotometer (ThermoFisher Scientific, Waltham, MA, USA). For normalization and internal control, 20 ng of total RNA was used per reaction in all qPCR. Both positive control (RNA from purified PPRV isolate) and negative control (nuclease-free water) were used simultaneously in all qPCR. The reaction was performed in ABI 7500 Fast Real-Time PCR system (Applied Biosystems, Foster City, CA, USA) using RevTrans QPCR One-Step EvaGreen (ROX) Kit (Bio-sell, Feucht, Germany). The following primer pairs were used: NrF1 5′-TGA CCA GGG AAG AAG TCA CA-3′ and NrR1 5′-TCG TCT TCA GGC ATG ATC TC-3′ to amplify a 120-bp product of N gene of PPRV (26). The cycle threshold (Ct) value for each reaction was recorded. Samples with Ct values of ≥40 were considered as negative.

### Antigen Detection in Tissues by Immunohistochemistry (IHC)

The paraffin sections of different organs from both PPRV-infected and control goats collected at 7, 10, 14, and 18 dpi were analyzed by immunohistochemistry for the localization of PPRV antigen. A monoclonal antibody against N protein of PPR virus (clone 38-4), received as a gift from IAEA, Vienna, Austria, was used as the primary antibody (12, 31). A commercial immunoperoxidase (IP) detection system, Dako EnVision® Dual Link System-HRP (DAB+) kit (Agilent Technologies, Santa Clara, CA, USA) was used to detect the bound primary antibodies. The paraffin sections were mounted on poly-l-lysine coated slides (Sigma, St. Louis, MO, USA). The IP staining was performed as described in the kit literature. With each group of staining, sections from the uninfected control group were also stained similarly to serve as the negative control.

### Statistical Analysis

Statistical analysis was performed using the software package GraphPad Prism Version 5.0. One-tailed non-parametric Mann–Whitney *U* test was used to calculate the statistical differences in hematological and biochemical profiles between PPRV-infected and healthy goats.

## Results

### Clinical Signs

Eight Black Bengal goats were challenged with a Bangladeshi strain of PPRV by the intranasal route and the clinical signs were recorded. The onset and types of clinical signs of individual goats are presented in Table 1. During the first 4 days post-infection (dpi), the infected goats showed no visible clinical signs. The clinical signs appeared between 5 and 8 dpi with mild depression, fever (40°C) and watery oronasal and ocular secretions. With time, the clinical signs became profound with fever, moderate depression and profuse watery nasal and ocular secretions. The clinical signs became aggravated during 10–14 dpi with further increase of body temperature (>41°C), purulent nasal and ocular discharges, some ulcerative lesions in the mouth and nostrils and severely inflamed eyelids. Severe diarrhea started during this phase of the disease. At 14 dpi and onward, the body temperature of the infected goats dropped down to subnormal temperature coupled with severe oral erosive lesions, profuse oronasal secretions, closed eyes due to sticky ocular discharges, severe dyspnea and severe diarrhea. Three out of eight infected goats died at 13, 15, and 18 dpi, the rest were sacrificed at different time points from 5 to 18 dpi (Table 1).

**Table 1:** Clinical signs and outcomes of animals following experimental infection.

**Goat ID**	**Onset of clinical signs**	**Clinical signs at onset**	**Clinical signs at advanced stage**	**Outcome**
**Infected (*****n =*** **8)**				
1	5 dpi	Ocular and oronasal secretion, fever (40°C)	-	Sacrificed at 5 dpi
2	5 dpi	Mild watery ocular and oronasal secretion, fever (40°C), mild dullness	Moderate oronasal and ocular secretion, fever (40°C), mild to moderate dullness and depression, mild anorexia	Sacrificed at 7 dpi
3	6 dpi	Ocular secretion, fever (40°C)	Moderate oronasal and ocular secretion fever (40.5°C), moderate dullness, anorexia	Sacrificed at 10 dpi
4	5 dpi	Mild ocular and oronasal secretion, fever (40°C), mild dullness	Profuse mucoid oronasal and ocular secretion, high fever (41°C), severe dyspnea, huge diarrhea, anorexia	Died at 13 dpi
5	7 dpi	Ocular and oronasal secretion, fever (40°C), mild dullness	Profuse oronasal and ocular secretion, high fever (41°C), severe dyspnea, diarrhea, anorexia	Sacrificed at 14 dpi
6	6 dpi	Mild ocular secretion, fever (40°C).	Profuse mucoid oronasal and ocular secretion, high fever (41°C), severe dyspnea, diarrhea, anorexia, subnormal temperature before death	Died at 15 dpi
7	7 dpi	Ocular and oronasal secretion, fever (40°C), mild dullness	Profuse oronasal and ocular secretion, high fever (41°C), severe dyspnea, diarrhea, anorexia, subnormal temperature before death	Died at 18 dpi
8	8 dpi	Ocular and oronasal secretion, fever (40°C)	Profuse mucoid oronasal and ocular secretion, high fever (41°C), severe dyspnea and diarrhea, diarrhea subsided after 16 dpi.	Sacrificed at 18 dpi
**Control (*****n =*** **4)**				
9	-	No clinical signs	No clinical signs	Sacrificed at 5 dpi
10	-	No clinical signs	No clinical signs	Sacrificed at 7 dpi
11	-	No clinical signs	No clinical signs	Sacrificed at 14 dpi
12	-	No clinical signs	No clinical signs	Sacrificed at 18 dpi

The cELISA showed that one goat was seroconverted as early as at 7 dpi with a competition percentage (CP) value of 22.59 (positive/negative threshold ≤ 35) and all the infected goats were seroconverted by 14 dpi with a CP value of 28.33 ± 7.33. All four control goats were healthy and seronegative throughout the experimental period.

### Pathological Changes

We studied the progressive development of gross and histopathological changes in the PPR infected goats. For this, we inoculated eight Black Bengal goats with PPRV, and sacrificed one infected goat at each time points of 5, 7, 10, 14, and 18 dpi. As controls, one uninfected goat was also sacrificed at 5, 7, 14, and 18 dpi. Besides, three of the infected goats that died at 13, 15, and 18 dpi were also examined and referred as “dead goats.” The gross and histopathological lesions in different organs of PPR infected goats at different days post-infection are recorded in Table 2.

**Table 2:** The presence and extent of gross and histopathological changes in different organs of PPRV infected Black Bengal goats.

**Organs**	**Sacrificed at**										**Died at**					
	**5 dpi**		**7 dpi**		**10 dpi**		**14 dpi**		**18 dpi**		**13 dpi**		**15 dpi**		**18 dpi**	
	**GP**	**HP**	**GP**	**HP**	**GP**	**HP**	**GP**	**HP**	**GP**	**HP**	**GP**	**HP**	**GP**	**HP**	**GP**	**HP**
Eyelids	–	+	–	+	–	+	+	++	++	+++	++	+++	++	+++	++	+++
Nostrils	–	–	–	–	+	+	++	++	++	++	++	++	++	++	++	++
Trachea	–	–	+	+	+	+	++	+	++	++	+++	++	+++	++	+++	++
Lungs	+	+	++	++	++	++	+++	+++	+++	+++	+++	+++	+++	+++	+++	+++
Lips	–	–	–	–	+	+	++	++	++	++	++	++	++	++	++	++
Oral mucosa	+	+	+	+	+	+	++	++	+++	++	+++	++	+++	++	+++	++
Tongue	+	+	+	+	+	+	++	++	+++	+++	+++	+++	+++	+++	+++	+++
Epiglottis	–	+	–	+	–	+	+	+	+	+	+	++	+	++	+	++
Esophagus	–	–	–	–	–	–	–	–	–	–	+	–	–	–	–	–
Rumen	–	–	–	–	–	–	–	–	–	–	–	–	–	–	–	–
Reticulum	–	–	–	–	–	–	–	–	–	–	–	–	–	–	–	–
Omasum	–	–	–	–	–	–	–	–	–	–	–	–	–	–	–	–
Abomasum	–	+	–	–	–	–	–	–	–	+	–	+	–	+	–	+
Duodenum	–	–	–	+	+	+	+	++	+	++	–	++	–	++	+	++
Jejunum	–	–	+	++	+	++	+	++	+	++	+	+++	+	+++	+	+++
Ileum	–	–	+	+	+	+	+	++	++	++	+++	+++	+++	+++	+++	+++
Cecum	–	–	+	+	+	++	++	+++	+++	+++	+++	+++	+++	+++	+++	+++
Colon	–	–	–	+	+	++	+	++	+	++	+	+++	+	+++	+	+++
Rectum	–	+	–	–	–	–	+	–	+	–	+	++	+	++	+	++
Heart	–	–	+	–	–	+	–	+	–	+	++	+	++	+	++	+
Liver	+	+	+	++	++	+++	+++	+++	+++	+++	+++	+++	+++	+++	+++	+++
Kidneys	+	+	+	++	+	++	++	++	++	+++	++	+++	++	+++	++	+++
UB	–	–	–	–	–	–	–	+	–	–	+	–	+	+	+	+
Spleen	–	+	–	+	+	++	++	+++	++	+++	++	+++	++	+++	++	+++
Tonsils	+	+	+	+	++	++	++	+++	+++	+++	+++	+++	+++	+++	+++	+++
PSLN	++	++	++	++	++	++	+++	+++	+++	+++	+++	+++	+++	+++	+++	+++
BLN	+	++	+	++	++	++	++	+++	++	+++	++	+++	++	+++	++	+++
MLN	–	–	+	+	+	+	++	++	++	++	++	++	++	++	++	++
Thyroid gland	–		–	–	–	–	–	–	–	–	+	–	–	–	–	–
Adrenal gland	–	–	–	–	–	–	–	–	–	–	+	–	–	–	–	–
Pancreas	–	–	–	–	–	–	–	–	–	–	–	–	–	–	–	–

*dpi, days post infection; GP, gross pathology; HP, histopathology; UB, urinary bladder; PSLN, prescapular lymph nodes; BLN, bronchial lymph nodes; MLN, mesenteric lymph nodes; Lesion scoring, –, absent; +, mild; ++, moderate; +++, severe*.

**Figure 1:**
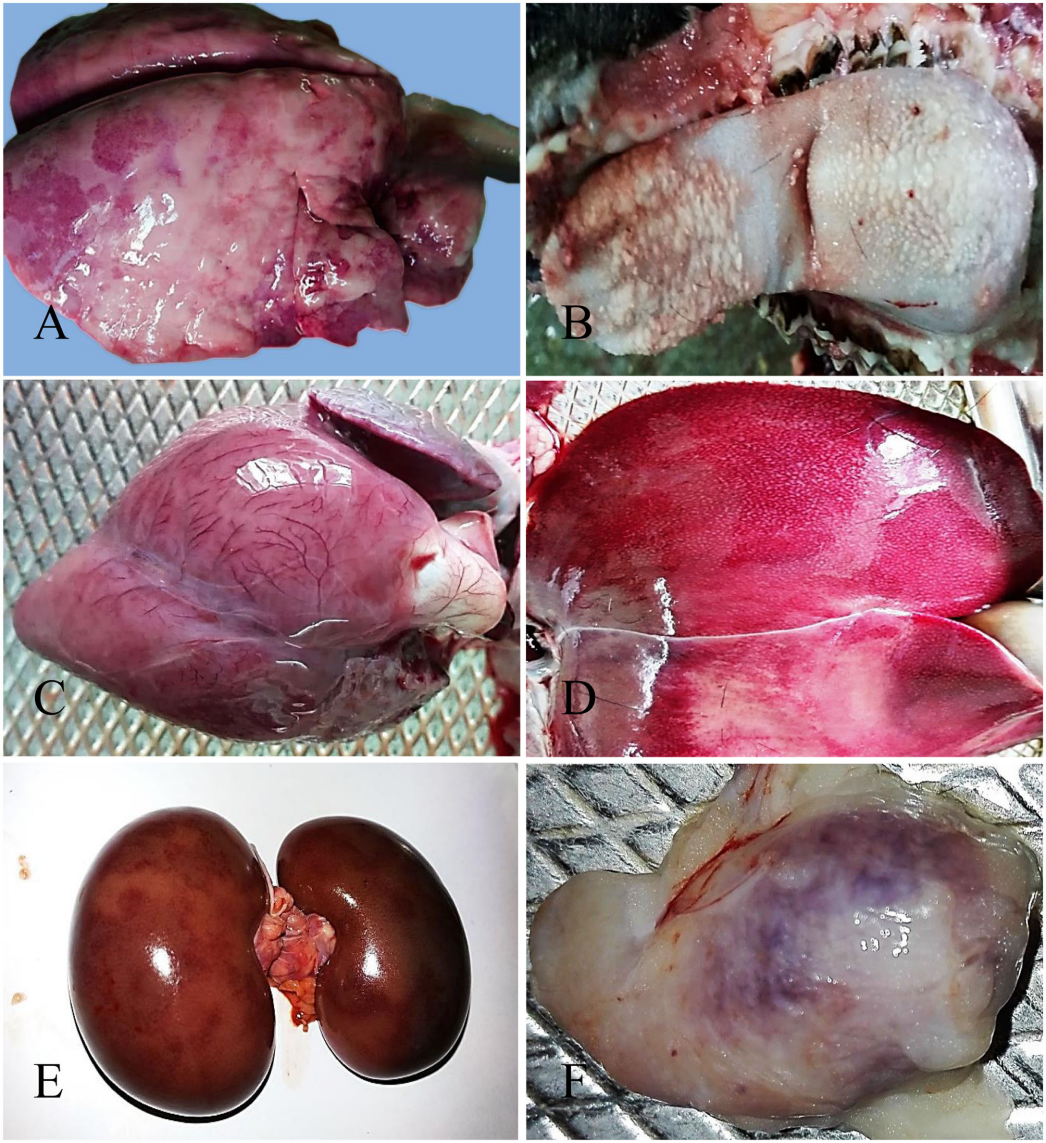
Gross pathological changes in the PPR infected Black Bengal goats. PPR infected goats showing **(A)** congestion, consolidation and fibrin deposition at 14 dpi, **(B)** necrosis and ulceration in the tongue at 18 dpi, **(C)** myxomatous degeneration of the coronary fat and congested myocardium in the heart at 15 dpi (dead goat), **(D)** congestion with whitish necrotic spots on the surface of the liver at 14 dpi, **(E)** congestion with severe hemorrhagic streaks on kidneys at 18 dpi (dead goat), and **(F)** congestion and hemorrhages in the tonsil at 5 dpi.

The nostril of PPR infected goats showed slight redness at 10 dpi, and erosive and necrotic changes with thick purulent nasal discharge were observed at 14 dpi and onward. The trachea showed hemorrhagic streaks at 7 and 10 dpi, which progressed rapidly with severe congestion and hemorrhagic spots and frothy mucous in between inter tracheal rings from 14 dpi. About 10–15% areas of the lungs (mostly apical lobe) showed congestion and consolidation at 5 dpi which progressed rapidly affecting 30–40% areas of lungs showing meaty appearance, focal congestion, petechial hemorrhages and fibrin deposition at 7 and 10 dpi. From 14 dpi, more than 70% areas of the lungs were affected with severe congestion, consolidation and fibrin deposition (Figure 1A). Taken together, the PPR infected goats showed progressive erosion and necrosis in the nasal passages and fibrinous pneumonia in the lung.

The lips of PPR infected goats showed slight redness at 10 dpi followed by erosive and necrotic changes coupled with thick purulent oral discharge at 14 dpi and onward. The tongue and oral mucosa showed mild redness at 5 dpi followed by necrotic changes at 7 dpi. The severity of the lesions increased gradually with the progression of the disease and there was sloughing of the epithelial layers of the tongue and oral mucosa after 14 dpi (Figure 1B). The stomach of PPR infected goats showed no significant gross changes at any time point. In the intestine, there was congestion and the lumen was filled with watery fluid at 5 dpi and 7 dpi. Hemorrhagic streaks and congestion were seen in the small intestine of PPR infected goats starting at 7 dpi. Marked hemorrhages and congestion were found in the ileum, cecum and colon at 10 dpi and the severity was increased at 14 and 18 dpi as well as in dead goats. To sum up, the PPR infected goats showed progressive erosion and necrosis in the upper alimentary tract and hemorrhages and congestion in the intestine.

In the heart, severe congestion was found in the myocardium of the three dead goats (Figure 1C). The liver showed thin fibrinous covering at 5 dpi followed by fragility, brownish discoloration with tiny necrotic spots, mild congestion and hemorrhagic streaks at 7 dpi (Figure 1D). With time, the severity of the lesions increased at 10 dpi. At 14 and 18 dpi, there were severe congestion, petechial hemorrhages and whitish necrotic spots on the surface of the liver. Brownish discoloration and hemorrhagic streaks were found in the liver of three dead goats. The gall bladder was also found to be distended in all PPR infected dead goats. Kidneys showed focal hemorrhagic spots at 5, 7, and 10 dpi. Massive hemorrhagic streaks, congestion and brownish discoloration of kidneys were found at advanced stages and in dead goats (Figure 1E). The urinary bladder of one dead goat (died at 13 dpi) showed hemorrhagic streaks in the inner surface of the organ. In summary, hemorrhages, congestion and necrotic spots were noticed in the liver of PPR infected goats starting at 5–7 dpi that progressed rapidly, whereas focal to massive hemorrhages and congestion were observed in the kidney.

Among lymphoid organs, tonsils (Figure 1F) showed moderate hemorrhages and swelling at 5 and 7 dpi with progressive congestion, inflammation and swelling observed at 10 dpi and onward. The retropharyngeal lymph nodes (RTLN) also showed congestion, hemorrhages, edema and swelling starting at 5–7 dpi and progressed rapidly. The prescapular lymph nodes (PSLN) became enlarged, severely hemorrhagic and congested at 5 dpi and the severity increased with time. The bronchial lymph nodes (BLN) were swollen, congested and showed hemorrhagic spots from 10 dpi. The mesenteric lymph nodes (MLN) showed mild congestion, hemorrhages and swelling at 7 and 10 dpi which intensified with the time at 14 and 18 dpi. The spleen showed enlargement and edema at 10 dpi which became pale, contracted and atrophied at 14 dpi and onward as well as in dead goats. Taken together, most of the regional lymphoid organs showed early onset of hemorrhages and inflammation between 5 and 7 dpi and the severity increased with time.

Other glandular organs such as the pancreas, thyroid gland and adrenal gland of the infected goats showed no or minimum changes at any time points post-infection. Similarly, no gross pathological changes were detected in goats from the control group at any time point.

**Figure 2:**
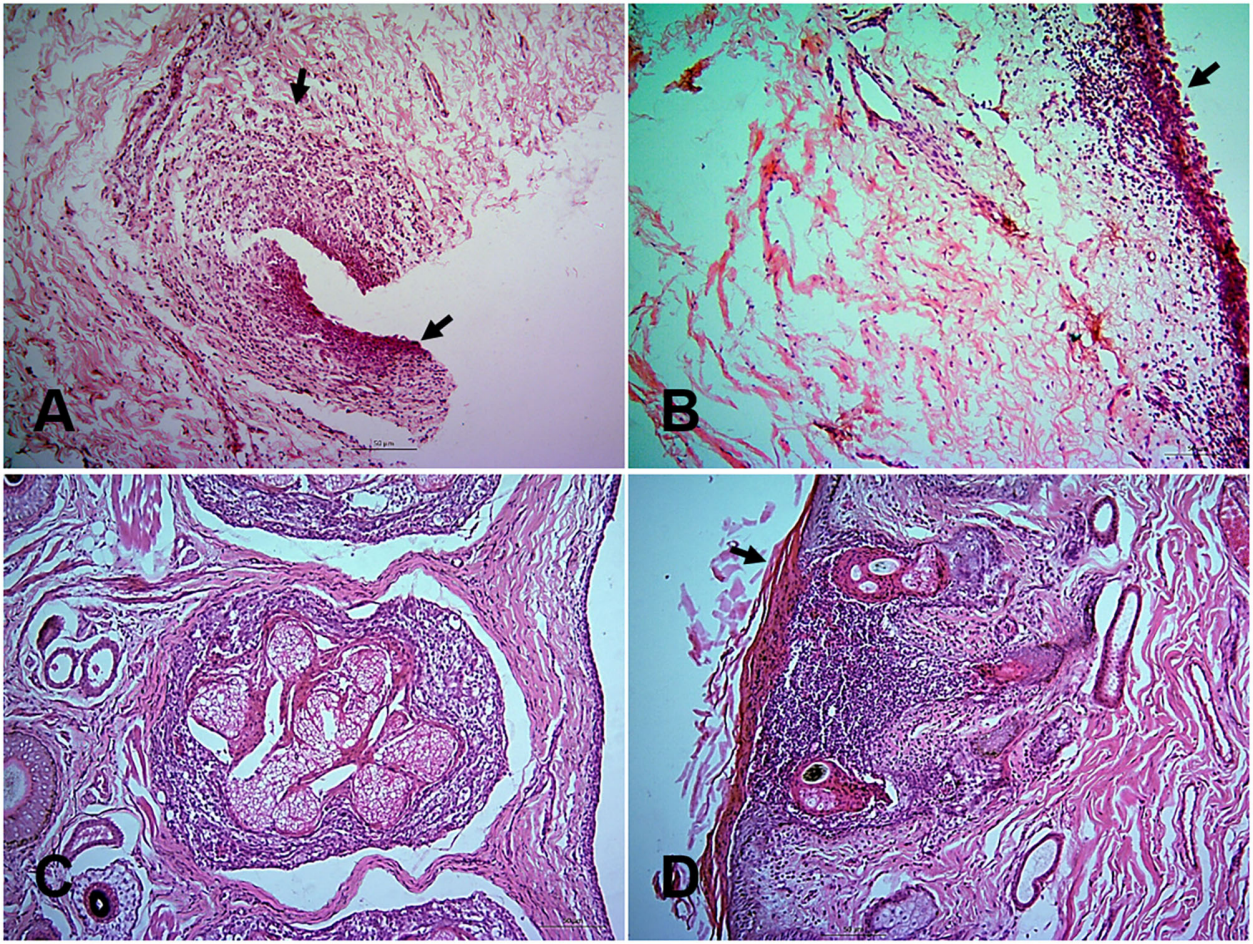
Histopathological changes in different tissues of PPR infected Black Bengal goats. Section of eyelids showing ulcerative conjunctivitis (arrows) at 10 dpi **(A)**, inflammation of eyelids (arrows) at 14 dpi **(B)**, pastular dermatitis, necrotic conjunctivitis with ulcerative lesions (arrow) in goats died at 13 **(C)**, and 15 **(D)** dpi. Bar = 50 μm. H&E stain.

At histopathology, PPR infected goats showed progressive conjunctivitis at 5, 7, and 10 dpi (Figure 2A). Blepharitis with mononuclear cells infiltration was seen in the eyelids of the infected goats throughout the study period (Figure 2B). Besides, severe congestion and hemorrhages were seen at 18 dpi. Chronic necrotic conjunctivitis with congestion, necrosis of lacrimal glands, mononuclear infiltration, pustular dermatitis with scalp formation, sloughing of the epithelial layer was found in the dead goats (Figures 2C,D).

**Figure 3:**
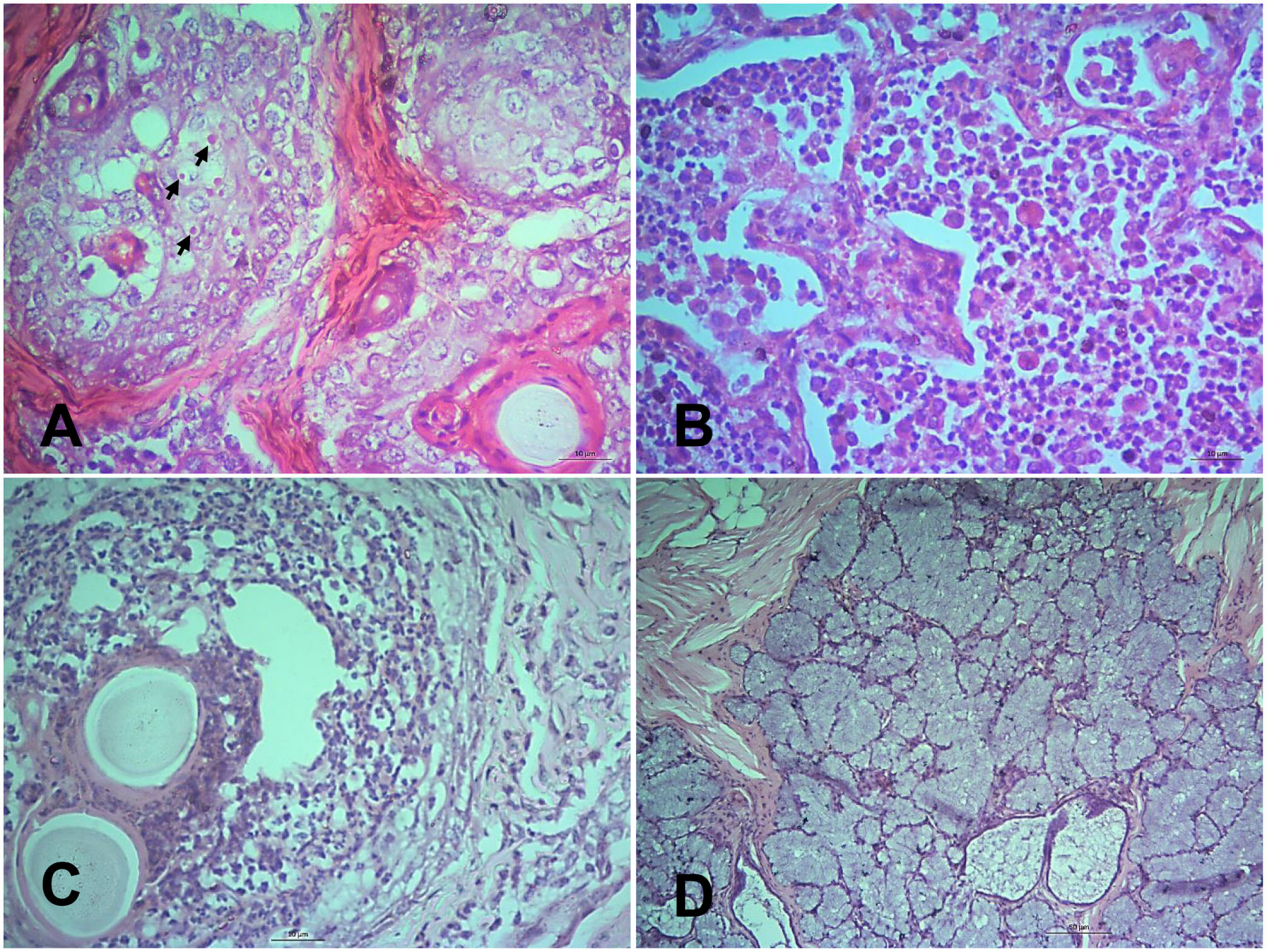
Histopathological changes in different tissues of PPR infected Black Bengal goats. Sections of tissues showing **(A)** ulcerative rhinitis, intracytoplasmic inclusion bodies (arrows), and necrosis of sebaceous gland at 14 dpi, Bar = 10 μm **(B)** severe mononuclear infiltration and syncytia formation in lungs at 7 dpi, Bar = 10 μm **(C)** vesicle formation with pyogranulomatous reactions in the lip at 10 dpi, Bar = 10 μm, and **(D)** necrotic salivary glands filled with copious mucus at 10 dpi, Bar = 50 μm. H&E stain.

In the nostrils and nasal passages, no distinct changes were observed at 5, 7, and 10 dpi. Hemorrhages, mononuclear infiltration, necrosis of sebaceous gland, and progressive ulcerative rhinitis (Figure 3A) with intracytoplasmic inclusion body in secretory glands were found at 14 and 18 dpi. In the trachea, mononuclear infiltrations, sloughing of the tracheal epithelium with hemorrhages in the lamina propria were seen at 7, 14, and 18 dpi and in dead goats. The first lesions in the lungs started with slight congestion, focal accumulation of mononuclear inflammatory cells at 5 dpi. In course of time, the alveoli ruptured and collapsed with massive infiltration of large mononuclear cells in the interstitial space and luminal sites at 7 dpi (Figure 3B). In addition, pyogranulomatous pneumonia, severe hemorrhage and congestion, mononuclear infiltration, syncytia formation and degeneration of syncytial cells and accumulation of pneumocyte II cells were observed after 10 dpi and in the dead goats.

The lips showed ulcerative cheilitis and pyogranulomatous lesions at 10 dpi (Figure 3C). Severe mononuclear infiltration of inflammatory cells, sloughing off of epithelial cells, congestion, intranuclear and intracytoplasmic inclusion bodies were found in the lips of dead goats. Necrotic and ulcerative stomatitis were started at 10 dpi. Salivary glands filled with mucus (Figure 3D) were consistently found in all goats after 5 dpi and sialoadenitis was found at 14 dpi and onward. In the tongue, hyperkeratinization of outer surface of epithelial cells was seen consistently in the PPR infected goats. In addition, progressive ulceration and sloughing off of epithelial cells coupled with intracytoplasmic and intranuclear inclusion bodies were found at different dpi and in dead goats. The mucous glands of the epiglottis were found mucous filled from 5 dpi and congestion and intracytoplasmic inclusion bodies in the glandular cells of epiglottis were found in the dead goats. In the stomach, desquamation of mucosal epithelium and fusion of abomasal villi together with mononuclear infiltration in the mucosal epithelium tissue were found in two goats sacrificed at 5 and 18 dpi. In the intestine, the duodenum, jejunum and ileum showed loss of mucosal epithelium, fusion and shortening of villi, mononuclear inflammatory infiltration at 5 and 7 dpi and progressed rapidly. The cecum, colon and rectum showed proliferation of goblet cells, shortening and fusion of villi, hemorrhages, deposition of necrotic debris with mononuclear inflammatory cells in the glandular areas at 5 dpi and progressed rapidly.

**Figure 4:**
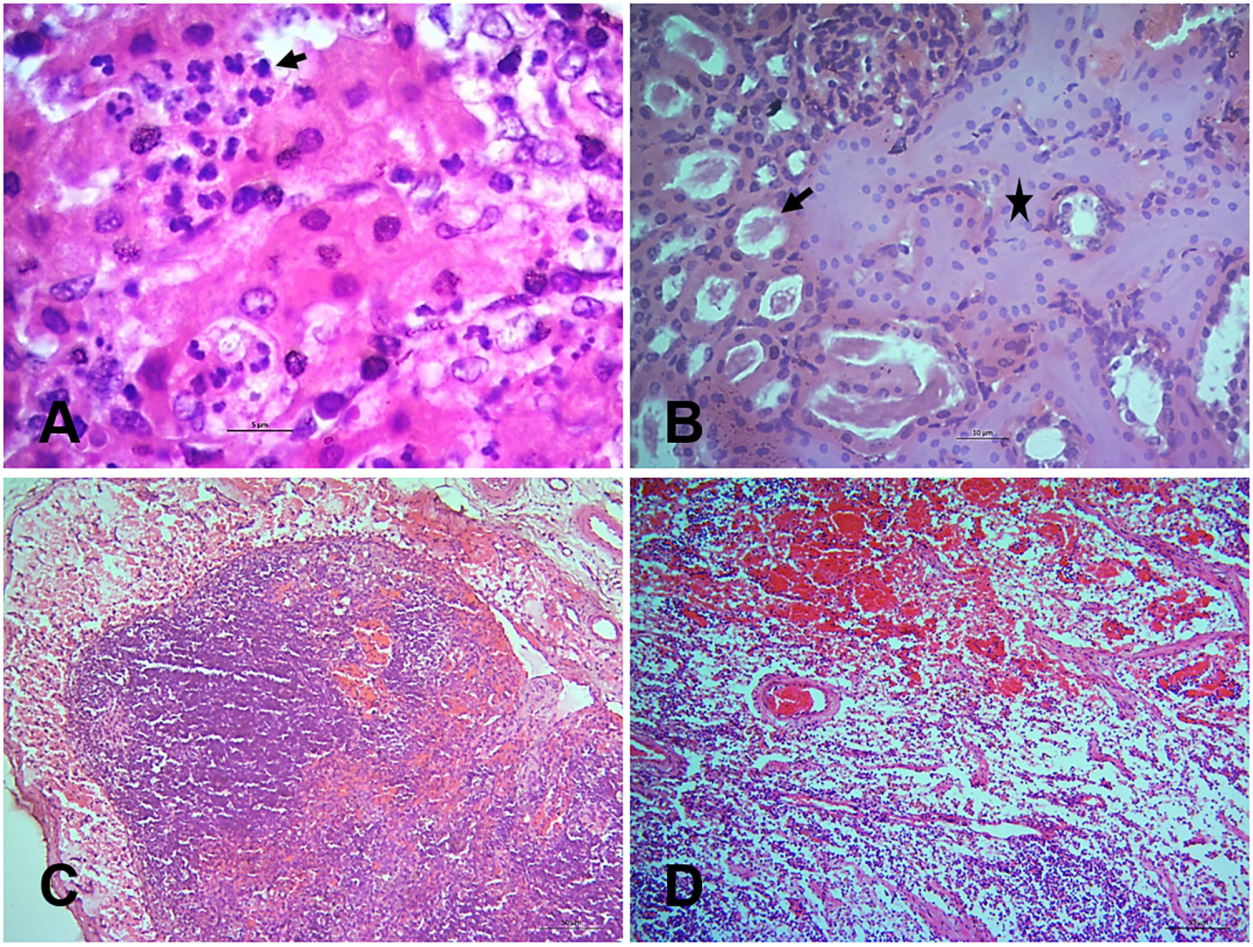
Histopathological changes in the tissues of PPR infected Black Bengal goats. Sections of tissues showing **(A)** hepatocellular necrosis with minute neutrophilic foci in the liver at 7 dpi (arrow), Bar = 5 μm **(B)** necrosis of renal tubular epithelium (arrow), collapse of renal tubules (asterisk), and glomerulonephritis at 10 dpi, Bar = 10 μm **(C)** lymphoid depletion with pericapsular hemorrhage in tonsils at 18 dpi, Bar = 50 μm and **(D)** severe hemorrhages, congestion with marked lymphoid depletion in the bronchial lymph node at 14 dpi, Bar = 50 μm. H&E stain.

In the heart hemorrhages, congestion and extravasation of RBC were observed after 10 dpi and also in dead goats. In the liver, fatty change and single hepatocellular necrosis with the infiltration of inflammatory cells predominantly neutrophils (Figure 4A) were observed at early stages (5 and 7 dpi). The single hepatocellular necrosis progressed to focal to multifocal necrosis. Widespread hemorrhages, fatty change and stagnation of bile, intranuclear, and intracytoplasmic inclusion bodies were found at advanced stages. Massive hemorrhages were found in kidneys at 5 dpi. At 7 dpi, there was necrosis of the renal tubular epithelial cells with swollen and fused tubular epithelial cells and hemorrhages (Figure 4B). Similar but more severe lesions were found in kidneys at 10 and 18 dpi and also in dead goats.

Among various lymphoid organs, tonsils (Figure 4C) showed slight lymphoid depletion with syncytia formation at 5 and 7 dpi, moderate lymphoid depletion at 10 dpi, lymphoid depletion with pericapsular hemorrhages at 14 and 18 dpi. The prescapular lymph node showed mild hemorrhages, congestion and lymphoid depletion at 5, 7, and 10 dpi. However, severe hemorrhages, congestion and moderate lymphoid depletion was found at 14 and 18 dpi as well as in the dead goats. The bronchial lymph node showed hemorrhages with moderate lymphoid depletion at 5, 7, and 10 dpi, severe hemorrhages, congestion with marked lymphoid depletion at 14 (Figure 4D) and 18 dpi. Hemorrhages, congestion and multifocal progressive lymphoid depletion were found in the mesenteric lymph nodes of the infected goats and the severity of the lesions was increased at 14 and 18 dpi. The spleen showed severe congestion, moderate hemorrhages, and focal lymphoid depletion at 5–7 dpi and progressed rapidly. In sum, lymphoid depletion of variable degrees coupled with hemorrhages and congestion were found in the lymphoid organs of PPR infected goats.

The control goats showed no histopathological changes in any tissues examined.

### Hematobiochemical Changes

We also examined the progressive changes in the hematological and serum biochemical parameters of PPR infected Black Bengal goats. To this end, we analyzed their different hematobiochemical parameters at 0, 5, 7, 10, 14, and 18 dpi. Leukocytosis in the infected goats was found at the first febrile phase of the disease, however, immediately after the initial febrile phase, leukopenia developed. On differential counts, lymphopenia and relative neutrophilia were noticed in the infected goats at 10 dpi and onward. The serum concentration of the total protein decreased significantly at 14 and 18 dpi. The concentration of the two important metabolites such as urea B and blood urea nitrogen also found significantly increased at 18 dpi. Similarly, the concentration of four important serum enzymes associated with kidney and liver functions such as CK, creatine kinase; ALP, alkaline phosphatase; AST, aspartate transaminase; ALT, alanine aminotransferase showed gradual elevation in the PPR infected goats. Among different electrolytes, the concentration of the sodium and chloride increased significantly in the infected goats. The detail of the hematobiochemical changes of experimental PPR infected goats has been published separately (32). Taken together, the PPR infected goats showed progressive leukopenia and hypoproteinemia, and a gradual increase of serum metabolites and enzymes associated with liver and kidney functions. Moreover, an imbalance in electrolytes (sodium and chloride) was also recorded in the infected goats.

### Evaluation of Virus Distribution in Different Tissues and Virus Shedding

**Figure 5:**
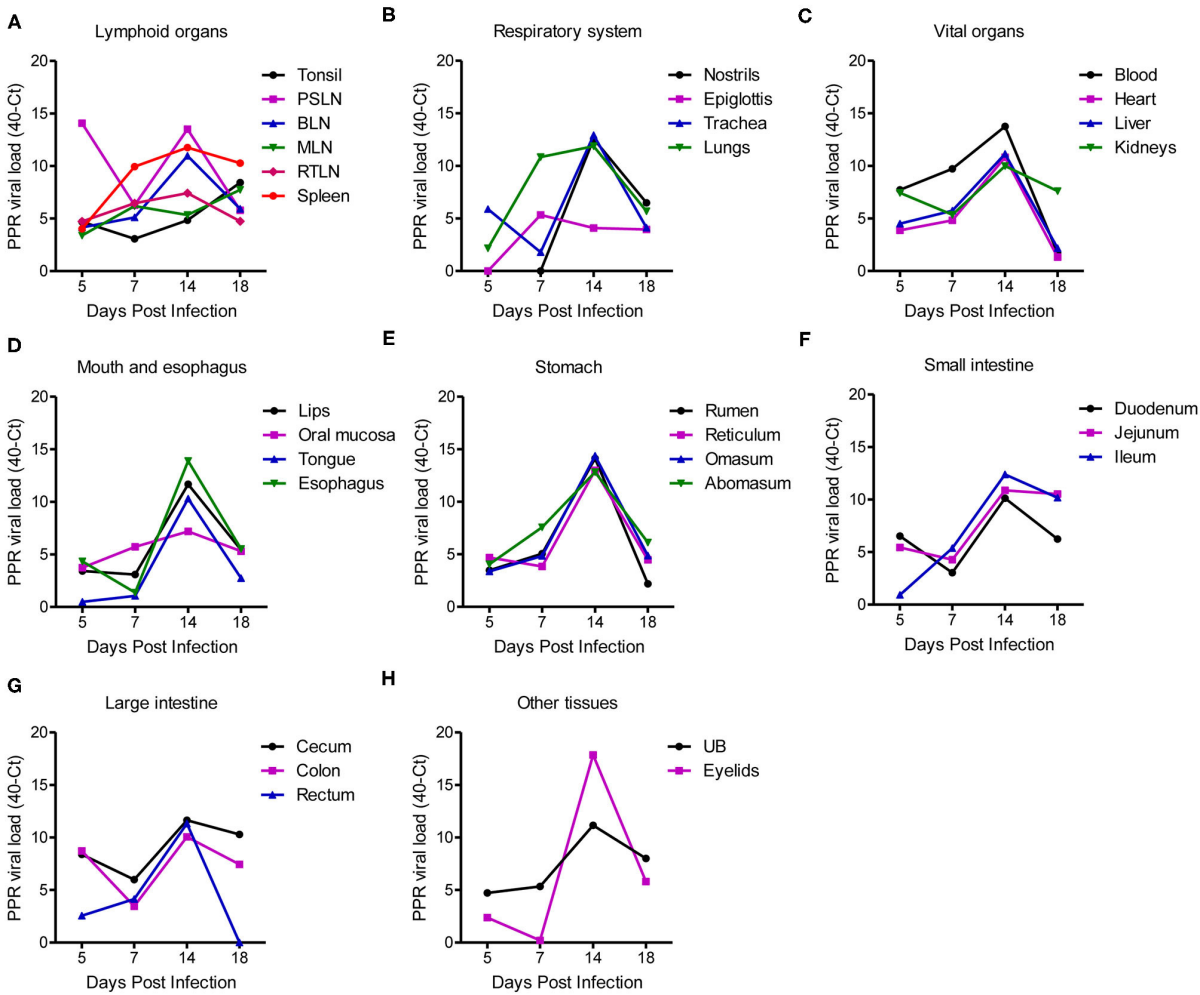
PPR virus loads in different organs and tissues of PPR infected Black Bengal goats expressed as 40-Ct. Tissues from PPRV infected goats were collected at different days post infection and the virus loads in **(A)** lymphoid organs, **(B)** respiratory system, **(C)** vital organs, **(D)** mouth and esophagus, **(E)** stomach, **(F)** small intestine, **(G)** large intestine, and **(H)** urinary bladder and eyelids were quantified. Ct, cycle threshold; PSLN, prescapular lymph nodes; BLN, bronchial lymph node; MLN, mesenteric lymph nodes; RTLN, retropharyngeal lymph nodes; UB, urinary bladder.

The quantity of the PPRV RNA was analyzed in different tissues of PPR infected goats sacrificed at 5, 7, 14, and 18 dpi as well as in three dead goats by real-time quantitative RT-PCR (qPCR). The viral loads in different tissues of PPRV-infected goats are expressed as 40-Ct and presented in Figure 5. A lower Ct value indicates a higher virus load in the sample. A relatively higher virus load was detected in all pharyngeal lymphoid organs at the early time point (5 dpi) and among them, the highest virus load was observed in the pre-scapular lymph nodes (Ct 25.92) at 5 dpi (Figure 5A). Other lymphoid organs such as bronchial lymph nodes and retropharyngeal lymph nodes showed a gradual increase of PPRV RNA from 5 dpi with a peak at 14 dpi and decreased thereby (Figure 5A). However, the tonsils, mesenteric lymph nodes and spleen maintained an elevated virus concentration up to 18 dpi. Lungs showed a moderate virus load (Ct 29.12) at 7 dpi with a peak (Ct 28.12) at 14 dpi (Figure 5B). The nostrils and trachea showed a sharp rise in virus load at 14 dpi with Ct values of 27.43 and 27.08, respectively (Figure 5B). Viremia started at 5 dpi (Ct 32.27) which increased gradually with a peak at 14 dpi (Ct 26.24) and declined afterward (Figure 5C). The heart, liver, kidneys showed detectable virus load at 5 dpi (Ct 32.55–36.12) which increased over time and peaked at 14 dpi (Ct 28.85–30.01) and reduced afterward, except kidneys which maintained a moderate virus load at 18 dpi with Ct value of 32.39 (Figure 5C). The organs of the digestive system (Figures 5D–G) showed low or moderate virus load during 5–7 dpi (Ct 32.43–39.51) with peak at 14 dpi (Ct 25.63–32.80). The virus load in these organs showed much reduction after 14 dpi except jejunum, ileum and cecum which maintained a relatively higher virus load (Ct 29.47–29.82) at 18 dpi. Among all tissues analyzed, the eyelids (Figure 5H) showed the highest virus load (Ct 22.14) at 14 dpi and decreased afterward. The adrenal gland and pancreas showed no detectable virus load at any time points analyzed. On the other hand, the three dead goats showed relatively higher virus load in most of the tissues at any time point compared to the sacrificed animals. In particular, the two goats that died at 13 and 15 dpi showed the highest virus load in different organs as compared to the sacrificed goats.

**Figure 6:**
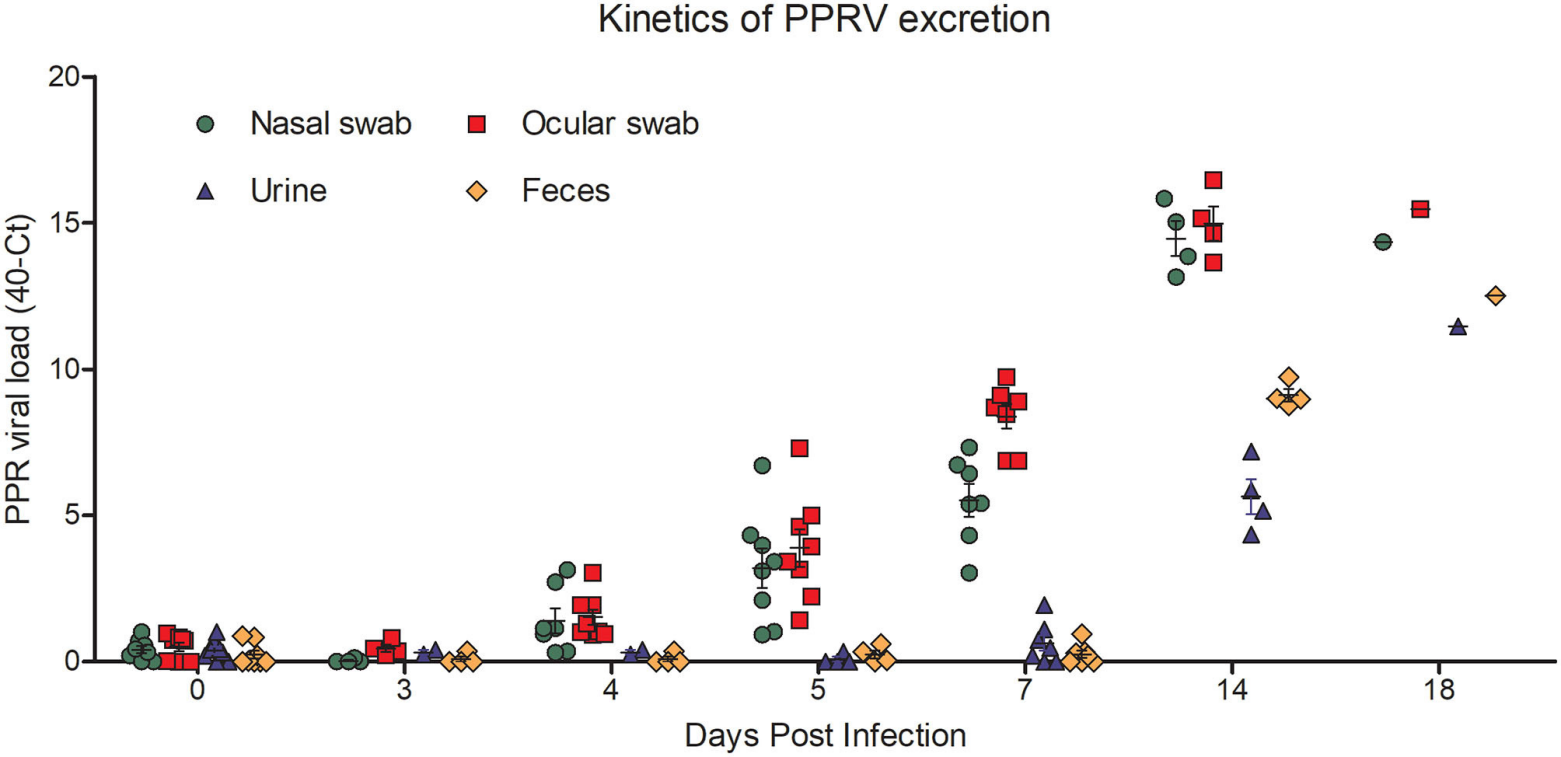
PPR virus shedding in ocular and nasal secretions and in the feces and urine of PPR infected Black Bengal goats. qPCR results are expressed as 40-Ct.

Next we quantified the amount of virus shedding in nasal and ocular secretions, as well as in urine and feces of PPR infected goats at 0, 3, 4, 5, 7, 14, and 18 dpi by qPCR. The nasal and ocular swabs of some infected goats showed detectable virus excretion as early as 4 dpi which increased slightly at 5 dpi. At 7 dpi, there was moderate virus excretion in nasal swabs (Ct 32.68–36.96) which increased continuously over time with a very high virus titer at 14 dpi (Ct 24.17–26.15) and 18 dpi (Ct 25.65) (Figure 6). Similarly, the ocular swabs showed moderate virus titer at 7 dpi (Ct 30.28–33.13) with peaks at 14 dpi (Ct 23.53–26.36) and 18 dpi (Ct 24.54) (Figure 6). On the other hand, a delayed kinetic was found for virus shedding in the urine and feces of the PPR infected goats than in the nasal and ocular secretions. Moderate virus shedding in urine was detected only at 14 dpi (Ct 32.85–35.65) which further increased at 18 dpi (Ct 28.53) (Figure 6). Similarly, the feces showed a moderate virus titer (Ct 30.26–31.25) at 14 dpi which increased at 18 dpi (Ct 27.49) (Figure 6). Taken together the qPCR analysis showed that virus shedding started earlier in nasal and ocular secretions (by 4 dpi) as compared to that in feces and urine (by 14 dpi), which gradually increased and continued till the end of the experiment (18 dpi).

### Immunohistochemical Localization of PPRV Antigen in Tissues

**Figure 7:**
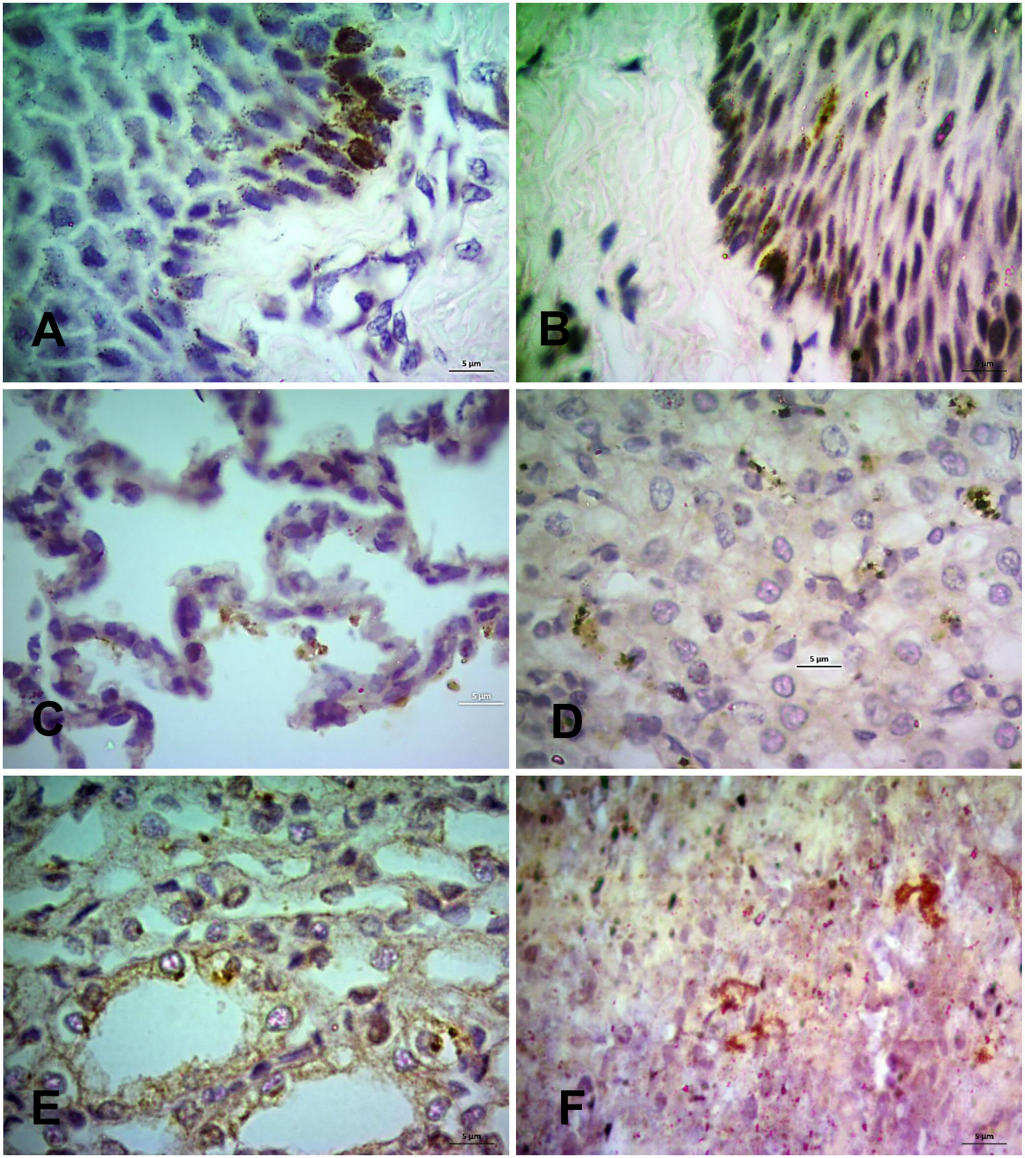
Immunohistochemical localization of PPRV antigen in different tissues of PPR infected Black Bengal goats. Sections of tissues showing PPRV antigens in **(A)** lips at 7 dpi, **(B)** tongue at 14 dpi, **(C)** lungs at 7 dpi, **(D)** liver at 10 dpi, **(E)** kidney at 10 dpi, and **(F)** prescapular lymph node at 7 dpi. IHC, Bar = 5 μm indicates magnification.

Finally, the PPRV antigen was detected in the formalin-fixed tissue sections of PPR infected goats at 7, 10, 14, and 18 dpi by immunohistochemistry (IHC) (Figures 7A–F). The IHC showed localization of PPRV antigen in different organs. Among various organs, the lips, tongue, nostrils, eyelids, lungs, liver, kidneys, spleen and tonsils showed positive reactions for PPRV antigen throughout the study period. However, epiglottis, lymph nodes, omasum, duodenum, jejunum, esophagus, ileum, cecum, colon, and rectum showed sporadic positive reactions at different time points.

## Discussion

Here we performed an in-depth sequential pathology study of a lineage IV Bangladeshi PPRV strain in Black Bengal goats. The study strain (BD/PPRV/2015/1) was originally isolated from a natural outbreak in Black Bengal goats having a high morbidity (80.44%) and mortality rates (65.67%) (10). For the experimental infection, we used the intranasal route of virus inoculation and all of the infected goats developed clinical PPR disease. At early time points (5–7 dpi), we detected hemorrhages and inflammation of the pharyngeal and retropharyngeal lymph nodes, tonsils and prescapular lymph nodes. Increased virus titers were also detected in these organs which indicated an initial virus replication. Of note, the highest virus load among lymphoid organs was detected in the prescapular lymph node at 5 dpi. This was due to the fact that similar to other morbilliviruses, the PPRV is received by the antigen-presenting cells/lymphocytes and epithelial cells of the nasal mucosa through the SLAM and Nectin-4 receptors, respectively, after intranasal inoculation (33–35). Virus-infected lymphoid cells then move to the local and regional lymph nodes through lymph flow and where the virus replicates, which was indicated by lymphoid depletion and hemorrhages in these organs. The early hemorrhagic and necrotic changes in the lymphoid tissues have also been reported previously (18). At 7 dpi, high virus titers were detected in the lungs, blood and spleen. Subsequently, the virus was also spread to other visceral organs (~5–9 dpi). Although the viremia was evident from 5 dpi with a gradual increase in the concentration of virus in blood till 14 dpi, it was not clear if the transportation of the virus to different organs was plasma or cell-associated. The replication of the virus resulted in moderate to severe gross and histopathological changes in the lungs of the infected goats characterized by hemorrhages, congestion, consolidation (30–40% of lungs), necrosis and syncytia formation at 7 dpi. Other visceral organs also showed mild to moderate pathology at this stage. The immunohistochemistry (IHC) also detected viral antigens in the lymphoid and epithelial cells of different organs. At this stage, infected goats showed high fever which could be due to viremia and associated inflammatory responses (20).

Following viremia, the virus reached the oral, nasal and ocular mucosa, and multiplied extensively, shedding the virus to the environment, and produced necrosis and ulceration with many intracytoplasmic and intranuclear inclusion bodies in the tissues. A large quantity of the virus was detected in the oral and nasal mucosa during this phase. The necrotic and ulcerative lesions in the oral and nasal mucosa might have been exposed to opportunistic bacterial infection, which further aggravated the lesions. Besides, at this advanced stage severe gross and histopathological changes were recorded in the trachea, lungs, gastrointestinal tract, lymphoid tissues, liver and kidneys. For instance, more than two-thirds of the infected lungs showed severe hepatization and fibrin deposition and goats had difficult open mouth breathing. These findings were consistent with some previous studies (10, 12, 18, 19). Of note, a very high virus titer was detected in the esophagus and stomach of the PPR infected goats, however, no or minimal gross and histopathological changes have been recorded in these organs. A similar phenomenon has been described in another recent study (18). In the intestine, mostly ileum, cecum and colon showed marked hemorrhagic and necrotic changes at 10–14 dpi, which intensified with time. A large quantity of PPRV was detected in these organs at this phase of infection and most of the affected goats showed severe diarrhea.

The liver and kidneys of the infected goats showed mild to severe pathological changes throughout the study period. Coagulation necrosis, fatty changes, hemorrhages and bile stagnation were the major pathological changes in the liver. PPRV was detected in the hepatocyte and inflammatory cells of the liver throughout the study. The hematobiochemical analysis showed an elevated level of aspartate transaminase (AST), alanine transaminase (ALT) and alkaline phosphatase (ALP) in blood at 10 dpi and onward, that mirrored the pathological changes in the liver. However, the fatty changes and bile stagnation in the liver were probably associated with starvation and liver inflammation, respectively. Hemorrhages, syncytia formation, massive necrosis and fusion of the renal tubules were found in the kidneys of the infected goats. PPRV is known to produce syncytia (fusion of cells) in Vero cells, a cell line derived from African green monkey kidney epithelial cells (36). The same phenomenon may have taken place in the infected kidneys in this study. PPRV antigen was also detected in the tubular epithelial cells of the infected goats. The elevated level of the creatine kinase (CK) in the blood also confirmed the renal injury. In addition, the tubular fusion and necrosis disrupted the kidney functions causing loss of protein (hypoproteinemia) and increased metabolite concentration in the blood such as blood urea nitrogen and urea B with the progression of the disease. Similar lesions in the liver and kidneys have also been found in the PPR infected Black Bengal goats from natural outbreaks (10) and have not been described in any of the earlier pathogenesis studies (8, 9, 14, 18).

After 14 dpi, the body temperature of the infected goats went down to a subnormal state due to diarrhea and multiple organ dysfunction. The infected goats started seroconverting at 7 dpi and all the surviving animals were seroconverted at 14 dpi. This was associated with a sharp reduction in the PPRV RNA in the blood. However, the nostrils, lungs, oral mucosa, intestine, kidneys and lymphoid organs (tonsils, spleen and mesenteric lymph nodes) showed a moderate virus titer, which led to a continuous shedding of the virus in natural secretions. As a matter, the nasal and ocular secretions of the infected goats carried a high virus titer at 14 and 18 dpi. Although there was minimal or no virus shedding in urine and feces at 7 dpi, a significant amount of virus shedding in urine and feces was observed at 14 and 18 dpi. As all of the infected goats died or were sacrificed at 18 dpi, further follow-up of the virus shedding could not be done. Further study should address the virus localization and shedding in the PPR infected goats that recover from a low dose infection. However, despite seroconversion, virus shedding continued. The recovered animals usually become immune against subsequent PPRV infection but could remain immunosuppressed until restoration of the lymphoid organs (37).

In conclusion, the lineage IV PPRV strain from Bangladesh produced severe lymphoid destruction at the early stage of infection in Black Bengal goats. However, with time, virus spread throughout the body and produced severe necro-hemorrhagic lesions in different visceral organs and also localized in the oral, nasal, ocular and intestinal mucosa leading to erosive and ulcerative lesions and shedding of the virus. All the infected goats developed serum antibodies by 14 dpi but virus shedding continued despite seroconversion.

## Data Availability Statement

The raw data supporting the conclusions of this article will be made available by the authors, without undue reservation.

## Ethics Statement

The animal study was reviewed and approved by Ethical Standard of Research Committee (ESRC), Bangladesh Agricultural University, Mymensingh.

## Author Contributions

EC: conceptualization, resources, supervision, project administration, and funding acquisition. SB: methodology. SB and MN: software and validation. SB, MN, MI, and EC: formal analysis and writing—review & editing. SB, MN, and EC: investigation, data curation, and visualization. MN and EC: writing—original draft preparation. All authors have read and agreed to the published version of the manuscript.

## Conflict of Interest

The authors declare that the research was conducted in the absence of any commercial or financial relationships that could be construed as a potential conflict of interest.
