# Supplementary material for: A Sequential Study on the Pathology of Peste Des Petits Ruminants and Tissue Distribution of the Virus Following Experimental Infection of Black Bengal Goats
Source: Front Vet Sci. 2021 Feb 19;8:635671. doi: 10.3389/fvets.2021.635671 (PMC7933573; doi:10.3389/fvets.2021.635671)
Supplement: Supplementary file 1 [file Table_1.DOCX]

Suppl. Table S1: Sequential histopathological changes in Black Bengal goats infected with PPRV

| Sl. No. | Organs | Days post infection (dpi) | | | | |
| --- | --- | --- | --- | --- | --- | --- |
|  |  | 5 dpi | 7 dpi | 10 dpi | 14 dpi | 18 dpi |
| 1. | Lips | No lesions | No lesions | Ulcerative dermatitis, pyogranulomatous lesion | Ulcerative stomatitis, sialoadenitis | Inflammation of lips (Cheilitis), ulcerative stomatitis |
| 2. | Nostril | No lesions | No lesions | No change | Ulcerative rhinitis, intracytoplasmic inclusion bodies in the secretory gland, necrosis of sebaceous gland | Ulcerative rhinitis, mucus gland filled with mucus, intracytoplasmic inclusion bodies, slight congestion |
| 3. | Oral mucosa | No lesions | No lesions | Necrosis in the salivary gland and full with mucus | Salivary gland filled with mucus | Stomatitis, salivary gland filled with saliva |
| 4. | Tongue | Sloughing off epithelial cells, keratinization of outer surface or epithelial cells of the tongue | Slight ulceration, deposition of keratin granules on the outer layer, presence of intracytoplasmic inclusion bodies | No lesions | No significant lesion | Severe ulceration, Sloughing off epithelial layer and mononuclear infiltration |
| 5. | Eye lids | Conjunctivitis, infiltration of mononuclear macrophages | Mononuclear infiltration, conjunctivitis | Mononuclear infiltration,  Chronic conjunctivitis | Blepharitis (Inflammation of eyelids), conjunctivitis | Severe congestion and hemorrhages |
| 6. | Epiglottis | Salivary glands of epiglottis were filled with mucus | No lesions | Salivary gland full with mucus (Multifocal) | Mucus gland filled with mucus | Salivary gland filled with saliva |
| 7. | Esophagus | Hemorrhagic lesion (Non-specific) | No lesion | No lesion | No lesion | No lesion |
| 8. | Trachea | Almost normal | Sloughing off tracheal epithelium, hemorrhagic lamina propria, presence of intranuclear inclusion | No change | Loss of tracheal epithelium and tracheitis | Severe congestion and hemorrhage |
| 9. | Lungs | Slight congestion, focal accumulation of some mononuclear inflammatory cells | 1. Collapsed and ruptured alveoli, interstitial space infiltrated with large mononuclear cells (massive),  2. Mononuclear inflammatory cells infiltration in the bronchioles and peri-bronchiolar space  3. Hemorrhages and degenerated bronchial epithelial cells deposition in the bronchiolar space,  4. Presence of free inclusion bodies and depletion of lymphocytes in the peri bronchial lymph node | Presence of granulomatous cells in lungs (pyogranulomatous pneumonia)  Presence of inflammatory cells within the bronchioles | Interstitial pneumonia, sloughing off bronchial or tubular lining/epithelium which deposits in the epithelial lumen (bronchial lumen), goblet cells proliferation, infiltration of mononuclear inflammatory cells in the lamina propria of the bronchi, broncho-interstitial pneumonia, severe acute hemorrhages in the alveolar space and rupture of the alveoli. Interstitial pneumonia with syncytia formation | Bronchioles filled with mononuclear inflammatory cells, interstitial pneumonia, severe hemorrhage with severe congestion, giant macrophages, and complete distortion of alveolar arrangement |
| 10. | Heart | No lesion | No lesion | No lesion |  | No lesion |
| 11. | Liver | Minute necrotic foci (about 20-25 micron), infiltration of inflammatory cells-specially neutrophils, multifocal necrosis in different hepatocytes, Degenerative change (cellular sickness)- more eosinophilic/acidophilic cytoplasm with hydropic change | 1. Majority of the cells with more eosinophilic cytoplasm, hydropic changes in hepatocyte, multi focal minute hemorrhages, 20% hepatocytes have pyknotic nucleus, stagnation of bile, diffuse necrotic hepatocyte, intranuclear and/or intracytoplasmic inclusion bodies, and fusion of hepatocytes/hepatic cells.  2. Severe mononuclear infiltration, minute to large coalescence multifocal necrosis, hepatocellular degeneration, also presence of minute neutrophilic foci  Multi focal and massive hemorrhage, hepatocytic necrosis. | Diffuse necrotic hepatitis, multi focal hemorrhage and congestion, excessive bile deposition  Diffuse hepatocellular necrosis, fatty change, and mononuclear inflammatory cells infiltration, Few hepatic cytoplasm become foamy and vacuolar. Few with eosinophilic cytoplasm, pyknotic nucleus,  Hemorrhages within the capsule with hemosiderin (p-1), chronic hepatitis with hemosiderosis, granuloma like inflammation in the liver (granulomatous hepatitis)  Total distortion of hepatic structure with massive necrosis, proliferation of the bile duct and bile stasis | Perihepatitis, multi focal hemorrhages and diffuse hepatocellular degeneration | Hydropic change with focal hemorrhage, more acidophilic cytoplasm, bile duct proliferation, pykonotic nucleus, congestion with hemorrhages, mononuclear infiltration within the central vein |
| 12. | Spleen | Severe congestions | Severe congestion with slight hemosiderosis | Slight lymphoid depletion | Slightly hemorrhagic and lymphoid depletion | Slight hemorrhage with focal lymphoid depletion |
| 13. | Kidneys | Massive hemorrhages | Massive necrosis in renal epithelial cells, presence of degenerative change in renal epithelium, fusion of tubular epithelial cells, closing of tubular lumen, tubular swelling, glomerulitis, hemorrhages, necrotic mass in tubular lumen. | Presence of edematous fluids within the tubular space, fusion of tubular epithelial cells (No tubules) with more acidophilic cytoplasm, Collapsed tubules and hyper cellularity within the globules (Multi focal)  Presence of intra cytoplasmic inclusion bodies | No lesion | Focal congestion with hemorrhages, tubules filled with necrotic mass |
| 14. | Rumen | Normal | No change | No change | No lesion | No lesion |
| 15. | Reticulum | No change/normal | No change | No change | No lesion | No lesion |
| 16. | Omasum | No change | No change | No change | No lesion | No lesion |
| 17. | Abomasum | Desquamation of mucosal epithelium, mononuclear infiltration in the mucosal epithelium tissue | No change | No change | No lesion | Focal fusion of abomasal villi |
| 18. | Duodenum | - | Loss of mucosal surface epithelium, fusion and shortening of villi, infiltration of mono nuclear inflammatory cells in the villi core and lamina propia | Loss of mucosal surface epithelium, fusion and shortening of villi, infiltration of mono nuclear inflammatory cells in the villi core and lamina propia | Slight duodenitis |  |
| 19. | Jejunum | Shortening of villi, fusion of villi, desquamation of villi, congestion of villi, mononuclear infiltration within villi | No change | Shortening of villi, fusion of villi, desquamation of villi, congestion of villi, mononuclear infiltration within villi | Slight jejunitis | Fusion and shortening of villi and sloughing off lamina epithelialis |
| 20. | Ileum | Normal | Ileitis, mononuclear infiltration, villi lining loss, fusion of villi and hemorrhage | Ileitis, mononuclear infiltration, loss of villi lining, fusion of villi and hemorrhage | Hemorrhagic ileitis | Ileitis, mononuclear infiltration, loss of villi lining, fusion of villi and hemorrhage |
| 21. | Cecum | Increase proliferation of goblet cells | Deposition of necrotic debris and mononuclear inflammatory cells infiltration in the cecal gland, Increase proliferation of goblet cells, shortening and fusion of villi | Transmural hemorrhage and congestion, lymphoid depletion, deposition of proteinaceous materials in the lymphoid follicles (Lymphoid necrosis) | Deposition of necrotic debris and mononuclear inflammatory cells infiltration in the cecal gland, Increase proliferation of goblet cells, shortening and fusion of villi | Shortening and fusion of villi Deposition of necrotic debris and mononuclear inflammatory cells infiltration in the cecal gland, Increase proliferation of goblet cells |
| 22. | Colon | Decrease no. of goblet cells | Fusion of villi and mononuclear inflammatory cells deposition | Deposition of necrotic debris and mononuclear inflammatory cells infiltration in the cecal gland, Increase proliferation of goblet cells, shortening and fusion of villi | Shortening and fusion of the villi, increase proliferation of goblet cells, accumulation of bloody exudate in the lumen | Shortening and fusion of the villi, increase proliferation of goblet cells, accumulation of bloody exudate in the lumen |
| 23. | Rectum | Desquamation of epithelium, shortening of villi and fused villi | No change | Fusion of villi and mononuclear inflammatory cells deposition | No lesion | No lesion |
| 24. | Tonsils | Slight lymphoid depletion, lymphangitis, necrosis of lymphocyte, infiltration of macrophages, presence of giant cells, presence of eosinophilic necrotic mass | Focal lymphoid depletion infiltration of large macrophages, mononuclear inflammatory cells infiltration | Lymphoid necrosis with lymphoid depletion within the lymphoid follicles | Pericapsular hemorrhage and lymphoid depletion | Severe lymphoid depletion with hemorrhages |
| 25. | PSLN | Hemorrhages and lymphoid depletion | Severely hemorrhagic and edematous | Lymphoid necrosis with lymphoid depletion within the lymphoid follicles | Lymphoid depletion | Severe hemorrhage and congestion, pericapsular lymphoid depletion |
| 26. | RTLN | Lymphoid depletion | Severe lymphoid depletion | Severe hemorrhagic and lymphoid depletion | Lymphoid depletion | Hemorrhages and lymphoid depletion |
| 27. | RPLN | Hemorrhages, lymphoid depletion and infiltration of mononuclear infiltration | Hemorrhages, lymphoid depletion and infiltration of mononuclear infiltration | Lymphoid depletion | Lymphoid depletion | Hemorrhages, lymphoid depletion and infiltration of mononuclear infiltration |
| 28. | BL | Lymphoid depletion and hemorrhagic | Lymphocytic depletion at the periphery of lymph node | Large mononuclear infiltration, Diffuse severe lymphoid depletion | Severe lymphoid depletion and severe hemorrhage | Large mononuclear infiltration, Diffuse severe lymphoid depletion |
| 29. | ML | Slight lymphoid depletion | Lymphoid depletion, slight hemorrhage and congestion | Lymphoid depletion, hemorrhage and congestion | Multi-focal lymphoid depletion | Lymphoid depletion, hemorrhage and congestion |
| 30. | Urinary bladder | No lesion | No lesion | Focal erosion of mucosal layer | No change |  |
| 31. | Adrenal gland | No lesion | No lesion | No lesion | No lesion | Slightly hemorrhagic |
