# Supplementary material for: A Sequential Study on the Pathology of Peste Des Petits Ruminants and Tissue Distribution of the Virus Following Experimental Infection of Black Bengal Goats
Source: Front Vet Sci. 2021 Feb 19;8:635671. doi: 10.3389/fvets.2021.635671 (PMC7933573; doi:10.3389/fvets.2021.635671)
Supplement: Supplementary file 2 [file Table_2.DOCX]

Supplementary Table S2. Histopathological changes in Black Bengal goats died of experimental PPRV infection

| Sl. No. | Organs | Histopathological findings in dead goats | | |
| --- | --- | --- | --- | --- |
|  |  | 13 dpi (Dead) | 15 dpi (Dead) | 18 dpi (Dead) |
| 1. | Lips | Associated salivary gland were congested | Severe mononuclear infiltration of inflammatory cells, sloughing off epithelial and mucosal layer of lips, ulcerative stomatitis, congestion, intranuclear and intracytoplasmic inclusion bodies | Sloughing of epithelial layer, ulcerative stomatitis, gland filled with mucus |
| 2. | Nostril | Ulcerative lesions, pastule formation, mostly mononuclear and neutrophilic infiltration, contamination of bacteria | Ulcerative rhinitis, mononuclear infiltration, sloughing off epithelium | Salivary gland filled with secretion/mucus, sloughing off keratinized epithelial layer |
| 3. | Oral mucosa | Salivary gland filled with mucus | Congestion, salivary gland filled with mucus, sloughing off epithelial layer | Congestion, salivary gland filled with mucus, sloughing off epithelial layer |
| 4. | Tongue | Necrotizing glossitis, hyper keratinization, necrotizing mononuclear infiltration, proliferation of basal cells of the tongue, intra nuclear inclusion bodies | Ulcerative pastular stomatitis, hemorrhages, giant macrophages infiltration, sloughing off epithelium | Necrotizing glossitis, hyperkeratinization, necrotizing mononuclear infiltration, proliferation of basal cells of the tongue, intranuclear inclusion bodies |
| 5. | Eye lids | Chronic conjunctivitis, necrosis in lacrimal gland, mononuclear infiltration, pastular dermatitis, scalp formation | Severe conjunctivitis, congestion, sloughing off epithelium layer, mononuclear infiltration | Slight congestion and secretory gland filled with secretions |
| 6. | Epiglottis | Mucus gland filled with mucus, intracytoplasmic inclusion bodies in the acinar cells | Salivary gland filled with mucus, congestion | Mucus gland filled with mucus |
| 7. | Esophagus | No lesion | No lesion | No lesions |
| 8. | Trachea | Only congestion | Congestion and sloughing off mucosal epithelium | Congestion and sloughing off mucosal epithelium |
| 9. | Lungs | Severe hemorrhage and congestion, mononuclear infiltration, syncytia formation and degeneration of syncytial cells, presence of giant macrophages, accumulation of pneumocyte II cells in the lumen of the alveoli | Aggregation of giant macrophages, severe congestion and hemorrhages, fusion of alveoli and filled with mononuclear inflammatory cells, mononuclear infiltration in the bronchioles | Severe congestion and hemorrhage, severe mononuclear infiltration |
| 10. | Heart | Congestion and slight extravasation of RBC | Severe congestion and hemorrhages | Mild congestion |
| 11. | Liver | More acidophilic cytoplasm, degeneration of hepatocytes, fibrinous layer on the outer surface, pyknotic nucleus, hemorrhages | More acidophilic cytoplasm, degeneration of hepatocytes, fibrinous layer on the outer surface, pyknotic nucleus, hemorrhages | Severe congestion, hydropic degeneration of hepatocytes, more acidophilic cytoplasm |
| 12. | Kidney | Multifocal severe hemorrhagic spots in the tubules of the medulla of kidneys, more acidophilic cytoplasm of renal epithelium, fusion of tubular epithelial cells, hypercellularity of the glomerulus | Severe congestion and hemorrhages, fusion of renal tubules | Severe hemorrhage in the tubular area, congestion, fusion of renal tubules |
| 13. | Spleen | Severely hemorrhagic and severe lymphoid depletion | Severe hemorrhages, lymphoid depletion, focal necrosis in the sinusoids | Hemorrhage and congestion, lymphoid depletion |
| 14. | Rumen | No lesion | No lesion | No lesion |
| 15. | Reticulum | Hemorrhage in one papillae | No lesion | Congestion and hemorrhages in the muscular layer |
| 16. | Omasum | No lesion | No lesion | No lesion |
| 17. | Abomasum | No lesion | No lesion | Congestion and fusion few of villi |
| 18. | Duodenum | No lesion | Sloughing off villi, fusion of secretory gland | Congestion, fusion and sloughing off villi |
| 19. | Jejunum | Severe congestion, shortening of villi, epithelial erosion, fusion and desquamation of villi | Severe fusion and desquamation of villi epithelium | Severely congested, fusion and shortening of villi |
| 20. | Ileum | Severe villus atrophy, necrosis in villi, collapsed and desquamation of villus epithelium, transmural hemorrhages and congestion, presence of proteinaceous deposition in the lymphoid follicles (called lymphoid necrosis) | Severe villus atrophy, necrosis in villi, collapsed and desquamation of villus epithelium, transmural hemorrhages and congestion, presence of proteinaceous deposition in the lymphoid follicles (called lymphoid necrosis) | Congestion, fusion and shortening of villi |
| 21. | Cecum | No lesion | Severe congestion, fusion and shortening and desquamation of villi epithelium | Severe congestion, fusion and shortening of villi |
| 22. | Colon | Shortening and fusion of villi, severe hemorrhage in the submucosa |  | Congestion, fusion of villi |
| 23. | Rectum | Slightly congested and proliferation of goblet cells | Fusion and shortening of villi, severe congestion | Sloughing off villi, shortening of villi and congestion |
| 24. | Tonsils | Hemorrhage slightly |  |  |
| 25. | Prescapular lymph node (PSLN) | Hemorrhage in the pericapsular and cortical region, slight lymphoid depletion | Congestion and lymphoid depletion | Severe hemorrhage and congestion, lymphoid depletion |
| 26. | Retrotracheal lymph node (RTLN) | Slight lymphoid depletion, multi focal hemorrhagic spot and congestion of cortical region | Lymphoid depletion, congestion at the peripheral region | No lesion |
| 27. | Retropharyngeal lymph node (RPLN) | Slight hemorrhage and slight lymphoid depletion | Severe lymphoid depletion, congestion and hemorrhages | No lesion |
| 28. | Bronchial lymph node (BLN) | Severe congestion, severe hemorrhage at the pericapsular region | Congestion and severe lymphoid depletion | Congestion, severe lymphoid depletion |
| 29. | Mesenteric lymph node (MLN) | Slight hemorrhage and slight lymphoid depletion | Lymphoid depletion and giant macrophages formation | Lymphoid depletion and giant macrophages formation |
| 30. | Urinary bladder | Congestion in the submucosa | Severely congested, hemorrhages, sloughing off columnar epithelium layer | No lesion |
| 31. | Adrenal gland | No lesion | Hemorrhages, slightly congested | No lesion |
| 29. | Thyroid gland | No lesion | No lesion | Full with thyroxin |
